# Surgery

**Published:** 1859-05

**Authors:** 


### SURGERY.

1. UPON THE CAUSES OF DEATH AFTER AMPUTATION. Read before the Royal Medical and Surgical Society.—By *Mr. Thomas Bryant, F.R.C.S.* The author, after having made a few preliminary observations, stated that his paper was based upon an analysis of three hundred cases of amputation, collected from the records of Guy's Hospital. He had divided them into four classes; and although he had not thought it necessary to alter the ordinary division of traumatic amputations into primary and secondary, he had made some change in the division of the other forms; for it became evident, in the analysis, that the classing together of such cases as amputations for talipes, tumors, elephantiasis, deformity, and others of a like character, with those of diseases of the joints, a wrong result must ensue; and, practically, this was found to be the case. He, therefore, divided these cases into pathological amputations and amputations of expediency, choosing the latter term as more accurately expressing the reason for the operation, as limbs are removed for tumor, talipes, elephantiasis, and deformity more from expediency than necessity; and he therefore suggested the use of such a term until a better was proposed. The author then proceeded to an analysis of the table of amputations, including one hundred and sixty-seven cases of pathological amputations, thirty-three amputations of expediency, seventy-six primary amputations, twenty-four secondary amputations; and having given in detail the analysis of each division, he summed up the whole in the following general conclusions:—1. That in amputations of the extremities, taken altogether, 25 per cent. are fatal; 30 per cent. in the lower extremity, and 10 per cent. in the upper. 2. That amputations, as a whole, are fatal in the following order:—Secondary, 50 per cent.; primary, 43 per cent.; amputations of expediency, 30 per cent.; pathological amputations, 12·5 per cent. 3. That in pathological amputations of the thigh, 18 per cent. are fatal, or 1 in 5·5; leg, 7·7 per cent are fatal, or 1 in 13·0; foot and upper extremity, success generally follows. 4. That in amputations of expediency of the thigh, 31·5 per cent. are fatal, or 1 case in 3·16; leg, 66·6 per cent. are fatal, or 1 case in 1·5; upper extremity fatal cases are exceptional. 5. That in traumatic amputations of the lower extremity, 60 per cent. are fatal; of the upper, 18 per cent.; and that traumatic amputations of the leg are, at least, as fatal as those of the thigh. 6. That secondary amputations are more fatal than primary. 7. That in amputation of the thigh for chronic disease of the knee-joint, 1 case only out of 7 proves fatal,* or about 14·5 per cent.; but for acute suppuration, a fatal termination is the rule. 8. That in amputations of the lower extremity for tumors, 36 per cent. are fatal; of the upper,

* The fatality of excision of the knee, according to Butcher, is at least one case in five.

recovery may generally be expected. The author then proceeded to the more immediate subject of the causes of death after amputations; and having given two tables showing the different causes of death, and their proportions or percentages both to the fatal cases and the whole number of amputations, he went on to the analysis of each division, and having given a detailed account of each, he condensed the whole into the following conclusions:—*General Conclusions upon the Causes of Death in Amputations Generally:* 1. That 25 per cent. are fatal; 30 per cent. of the lower extremity, 10 per cent. of the upper. 2. That pyæmia is the cause of death in 42 per cent. of the fatal cases, and in 10 per cent. of the whole number amputated. 3. That exhaustion is the cause of death in 33 per cent. of the fatal cases, and in 8 per cent. of the whole number amputated. 4. That the following causes of death are fatal in the annexed proportions:—

| | | Of Fatal Cases. | | | Of Whole Number. |
|---|---|---|---|---|---|
| Secondary hemorrhage, | | 7· | per cent., | or | 1·66 per cent. |
| Thoracic complications, | | 5·6 | " | | 1·33 " |
| Cerebral | " | 3· | " | | ·66 " |
| Abdominal | " | 1·4 | " | | ·33 " |
| Renal | " | 3· | " | | ·66 " |
| Hectic | " | 3· | " | | ·66 " |
| Traumatic | " | 7· | " | | 1·66 " |

*Pathological Amputations.*—1. That pathological are by far the most successful amputations, 12·5 per cent proving fatal. Such amputations of the upper extremity are generally followed by success. Of the lower extremity, 15 per cent. terminate fatally. 2. That pyæmia is the chief cause of death, proving fatal in 43 per cent. of the fatal cases, and in 5·4 per cent. of all pathological amputations; and when fatal, as a rule, it causes death within fourteen days of the operation. 3. That exhaustion, either from the shock of the accident or of the operation, from hemorrhage, or all three causes combined, is the cause in 33 per cent. of the fatal cases, or 4 per cent. of all amputations. 4. That secondary hemorrhage is the fatal cause in only 9 per cent. of the fatal cases, and in 1·4 per cent. of all amputations. 5. That hectic, abdominal, and thoracic complications act equally as causes of death in 13 per cent. of the fatal cases, and in 2 per cent. of all amputations. *Amputations of expediency:* 1. That 30 per cent. are fatal; but as amputations of the upper extremity are, as a rule, successful, the percentage of this operation upon the lower is much increased, 40 per cent. proving fatal. 2. That pyæmia is the chief cause of death, proving fatal in 60 per cent. of the fatal cases, and in 18 per cent. of all such amputations; and when fatal, as a rule, death takes place within fourteen days of the operation. 3. That death from exhaustion occurs in but 10 per cent. of the fatal cases; and that some thoracic or renal complication, or carcinomatous infiltration, are fatal causes in the same proportion. *Primary Amputations:* 1. That 43 per cent. are fatal; 60 per cent. of the lower extremity, and 30 per cent. of the upper. 2. That primary amputations are more successful than secondary. 3. That pyæmia is the cause of death in 43 per cent. of the fatal cases, and in 16 per cent. of the whole number, and that when fatal, the symptoms appear, as a rule, between the seventh and fourteenth days after the operation, and cause death in the third or fourth week, and not during the first two weeks, as in pathological amputations and those of expediency. 4. That exhaustion is the cause of death in 32 per cent. of the fatal cases, and in 12 per cent. of the whole number. 5. That traumatic complications prove fatal in 15 per cent. of the fatal cases, and secondary hemorrhage, cerebral or thoracic complications, about 7 per cent. each; renal disease proving a cause of death in 3·5 per cent. *Secondary amputations:* 1. That 50 per cent. are fatal; 68 per cent. of the lower extremity, and 12·5 per cent. of the upper. 2. That secondary amputations are more fatal than primary, by about 8 per cent. 3. That exhaustion is the chief cause of death, proving the cause in 60 per cent. of the fatal cases. 4. That pyæmia is the cause in 25 per cent. of the fatal cases; secondary hemorrhage and hectic in the remaining 15 per cent. *Conclusions upon Pyæmia as a Cause of Death:* 1. That it is the cause of death in 42 per cent. of all fatal cases of amputations, and in 10 per cent. of all amputations. 2. That it is the cause of death in the different forms of amputation in the following order: 1. In 70 per cent. of all fatal amputations of expediency. 2. In 43 per cent. of all fatal primary amputations. 3. In 43 per cent. of all fatal pathological amputations. 4. In 25 per cent. of all fatal secondary amputations; and that in amputations of expediency it is the most frequent cause, and in secondary amputations the least. 3. That in amputations for acute suppuration of the knee-joint, whether the result of an abscess discharging into the joint or otherwise, pyæmia is a more frequent cause of death than in amputations for chronic disease. 4. That it is the general cause of death in amputations for talipes, elephantiasis, and tumors. 5. That in primary amputations, and in amputations of expediency of the leg, it is a more frequent cause of death than in the same operations upon the thigh. 6. That, upon the whole, pyæmia appears to be a more frequent cause of death in amputations through limbs, the tissues of which are in a normal condition, and where a large surface of healthy bone is exposed. 7. That in pathological amputations, and in amputations of expediency, pyæmia, as a rule, proves fatal within fourteen days; but after traumatic amputations, the period of death is about the twenty-fifth or twenty-sixth day. *General Conclusions upon Amputations of the Thigh:* 1. That 27 per cent. are fatal. Pathological amputations, 18 per cent.; amputations of expediency, 31 per cent.; primary amputations, 60 per cent.; secondary, 75 per cent. 2. That in amputations of the thigh for chronic diseases of the knee-joint, about 15 per cent. are fatal, or 1 case in 7. 3. That amputations of the thigh for acute suppuration in the joint are generally fatal; and that pyæmia is the chief cause of death in these cases. 4. That exhaustion and pyæmia are causes of death in equal proportions, or in about 40 per cent. of the fatal cases, and in 10 per cent. of all amputations of the thigh. 5. That exhaustion is most fatal in primary amputations, and the least so in amputations of expediency. 6. That pyæmia is most fatal in amputations of expediency, and the least so in primary. 7. That primary amputations are, for the most part, fatal from exhaustion, 35 per cent. of the cases sinking from this cause, 15 per cent. from pyæmia, and secondary hemorrhage and traumatic complications, 5 per cent. each. 8. That exhaustion, pyæmia, and hectic are equally fatal causes in secondary amputations, proving fatal in 25 per cent. each. *Amputations of the Leg:* 1. That 37 per cent. are fatal; pathological amputations, 7·7 per cent.; amputations of expediency, 66·6 per cent.; primary amputations, 62·5 per cent.; secondary amputations, 66·6 per cent. 2. That amputations of the leg are 10 per cent. more fatal than of the thigh; the amputations of expediency and traumatic amputations being more fatal, and the latter more frequent. 3. That amputations of expediency of the leg are generally fatal, being twice as fatal as those of the thigh; that pyæmia is the chief cause of death in 75 per cent. of the fatal cases, and in 50 per cent. of all such amputations. 4. That in primary amputations, pyæmia is the cause of death in half the fatal cases, or in 32 per cent. of all such operations; exhaustion and visceral complications about 8 per cent. each. 5. That comparing primary amputations of the thigh and leg together, they are equally fatal; but that pyæmia is twice as fatal in amputations of the leg as in amputations of the thigh. 6. That half the cases of secondary amputations die from exhaustion; pyæmia and secondary hemorrhage being fatal in 8 per cent. each. 7. That taking all amputations of the leg together, 42 per cent. of the fatal cases die from pyæmia, and 32 per cent. from exhaustion. *Amputations of the Upper Extremity:* 1. That 10 per cent. are fatal. 2. That pathological amputations and those of expediency are, as a rule, successful. 3. That about 20 per cent. of traumatic amputations are fatal; 22 per cent. of the arm, and 16 per cent. of the forearm. 4. That one-third of these fatal cases die from pyæmia; one-third from some traumatic complication; and the remaining third from secondary hemorrhage or visceral disease.

Mr. Solly thought no one would dispute the utility of the paper. The statistics contained in it, however, extended over a great length of time, and referred more to the treatment of our ancestors than of the present generation. They must bear in mind that many of those cases which were said to be fatal after amputation, would be fatal independently of that operation; and although the amputation gave the patient a chance of life, yet that was all that could be said for it in such cases. It had been the rule in the hospitals with which he had been connected, never to make an amputation during an acute condition of the joint, as, unless that could be subdued, a fatal result might be expected. Some of the author's facts appeared to indicate that resection of the joint might often be adopted in preference to amputation.

Mr. Spencer Wells observed that, in a large proportion of the cases reported by the author, pyæmia appeared to have been the cause of death. He would like to ask, whether in Guy's Hospital the deaths from this cause were more frequent in the new than in the old buildings? The method of ventilation in the new portion of that hospital was not one which he should like to see generally carried out. The hospital-smell was very oppressive, and the air was hot and dry—conditions which he considered likely to favor the increase of pyæmia. In Paris, at the Necker Hospital, there were some old-fashioned buildings, with small rooms, dark, and not well ventilated; not at all the kind of buildings one would think desirable for a hospital. Subsequently they had erected some new buildings in connection with that hospital, constructed on the most approved modern principles, as regards ventilation, heating, lighting, etc., and with much larger wards, similar to the new rooms at Guy's. But it was shown, a few years ago, by M. Trousseau, that the mortality was greater in the new buildings than in the old. This was explained partly by the artificial system of ventilation, and partly by the fact of so many patients being in the same ward. He (Mr. Wells) would like to know if that was the case in the new portion of Guy's Hospital. He did not know whether operation cases had been put into the new building or not; but he thought the old buildings preferable to the new. He would like to know, too, how far the modes of wet and dry cleaning could be brought to bear upon the number of deaths from pyæmia, and what influence was produced by proximity to the dissecting-rooms. He had been informed, when at Strasburg, a few months ago, that at the hospital there the mortality from pyæmia was very considerable in those wards which were immediately over the dissecting-room; and that after removing all operation cases to the opposite side of the building, pyæmia had almost disappeared. This fact, he believed, might throw some light upon the same class of cases in English hospitals, and might justify the objection which the governors of our old hospitals had to the proximity of the dissecting-rooms to hospitals.

Mr. Birkett said that they had had no surgical cases in the new wards at Guy's Hospital at present, except some trifling operations; and pyæmia cases were almost unknown in them. Death had certainly taken place from pyæmia in one or two cases, but they were cases of simple wounds. It was a great pity, he observed, that the authorities of the different hospitals in London did not come to some arrangement to prevent the putting of surgical cases in the same ward as those where pyæmia cases had been placed. He could not help wishing also that they had better sanitary arrangements in the metropolitan hospitals, and that the governors of hospitals would consult their medical officers more frequently upon such arrangements.—(*London Medical Times and Gazette*, March 5, 1859.)

2. ON MANUAL COMPRESSION IN THE TREATMENT OF ANEURISM AND INFLAMMATION. By *Professor Vanzetti*. Dr. Vanzetti, Professor of Clinical Surgery at Padua, observes, in a recent communication addressed to the "Académie des Sciences," that although compression of the artery by the hand has occasionally been employed adjuvatory to other means of treating aneurism, yet this simple and natural method had never been proposed as the sole means of treatment, until he himself resorted to it. Since his visit to Dublin, in 1843, when he had the opportunity of observing the inconveniences attendant upon the production of compression by the aid of mechanical appliances, he has had in view the desirableness of substituting the action of the hand. It was not until 1853, however, that he first practically realized the idea. A mason presented himself with a popliteal aneurism the size of an orange, and after he had been prepared, by rest, diet, and nitre drinks, he was taught how to compress the femoral artery, and was directed to do this several times in the day. At the end of a fortnight, the compression was kept up more methodically, being continued, at intervals, so as not to weary the patient, for at least two hours at a time. Solidification then took place at the end of forty-eight hours. The tumor disappeared rapidly, and the man has continued well since. The second case occurred in the person of an officer of the Chasseurs, the popliteal aneurism being as large as a lemon. Having employed occasional compression of the femoral at home at intervals during a month, he came to the hospital, and then five hours' continuous compression, kept up by the pupils, secured solidification. He is now on duty in the army. 3. A woman, aged 38, was admitted into the Padua Clinic, with a frightful protrusion of the left eye, this having originally commenced during the throes of labor. As all the symptoms of aneurism of the ophthalmic artery were present, compression of the left carotid was put into force. The intervals were very short, for if continued for more than a minute, the pressure produced faintness. After four days all sounds and pulsations had ceased, and a few days later the eye reassumed all its normal characters. 4. The next case was an aneurism at the bend of the elbow, under the care of Professor Gherini, of Milan; and manual compression of the brachial produced solidification in three and a half hours. 5. Dr. Gelmi, of Verona, treated a case of popliteal aneurism by means of pressure of the femoral, first for two or three hours, and then for one or two hours per diem; and by the twentieth day the leg had resumed its normal condition. 6. A patient came under Professor Riberi's care at Turin, with an aneurism at the lower third of the femoral, and which had ensued on a fall. Four hours' compression produced the necessary solidification. 7. In the case of a woman who suffered from protrusion of the eyeball, dependent on aneurism, compression was made on the carotid during five minutes five or six times in the twenty-four hours. On the seventeenth day, and after 440 minutes of compression, all appearance of projection had been removed. The reason that indirect manual compression has not excited earlier attention, as a means of treating aneurism, is probably the idea that it required very long continuance. The cases related, however, prove that the time is in reality very short, and the compression being employed intermittingly, is attended with neither pain nor danger.

Encouraged by his success in the treatment of aneurism, Professor Vanzetti has resorted to manual compression in the case of inflammation of the limbs, whenever the artery has been accessible; and such success has attended its application to the femoral, brachial, and subclavian arteries in phlegmon and arthritis, that it now forms the ordinary means of treating such cases at the Padua Clinic whenever it is practicable. Employed promptly, it soon may arrest the progress of the inflammation, more time being required when the process has been set up during some period. In the case of severe or much advanced inflammation, although not of itself competent to effect a cure, it is the most powerful adjuvatory means. In the face of the great advantages derivable from this means, we must not be deterred by apparent difficulties in the execution. Persons can be generally got to make the compression, or the patient may be taught to do this; and, in a case of great urgency, the surgeon himself should perform it for two or three hours. In general, compression need only be kept up for eight or ten minutes, and, after resting, again resumed. The professor relates two cases as remarkable examples of the efficacy of this treatment: one being an instance of a bad phlegmonous erysipelas of the arm, treated by compression of the subclavian; and the other, a case of acute arthritis of the wrist, treated by compression of the brachial.—*Comptes Rendus*, t. xlvii. pp. 471–5, and *L'Union Méd.* 1858, No. 58, No. 115.

*The Annali Universali de Medicina* for July, 1858, contains notes of two additional cases of aneurism of the ophthalmic artery treated by digital compression with success. One of these is related by Dr. Gioppi, Professor of Diseases of the Eye, at Padua, and the other was communicated by Dr. Scaramuzza, of Verona, to Dr. Vanzetti, for publication. Dr. Vanzetti appends to the case the following summary of the general subject of digital compression:—1. The dominant principle of the treatment is that the compression should be complete and intermitting. 2. Complete compression, when made by the finger, is not painful. 3. Complete compression, made during a given time, produces an immensely greater effect than does incomplete compression continued for a like period. 4 Incomplete compression may, however, be resorted to when any local or general circumstances do not allow of its being made completely. 5. The compression may be intermittent, for being complete, it is powerfully efficacious in effecting solidification, even when continued for a brief space of time at short intervals. 6. The person employing it should continue it until his hand has need of rest, and he should resume it when this has been obtained, discontinuing it during the night, in order that the patient should enjoy his sleep undisturbed. 7. The patient can compress for himself the femoral, radial, branchial, carotid, and frequently the subclavian, and can continue it for about five to eight minutes. 8. The rapid cures obtained by Gherini and Riberi by means of compression continued uninterruptedly for a few hours, have not been attended with any accident. 9. The causes why an aneurismal tumor, which has ceased to pulsate after continuous compression for some hours, may, perhaps, as happens sometimes after the ligature, in place of solidifying, inflame, suppurate, or become gangrenous, are not as yet well known, and call for further study. 10. It is possible that the cruor does not become solidified, or softens in consequence of a pre-existing morbid condition of the sac, or owing to the disturbance of the capillary circulation of its parietes; and that the inflammation and subsequent gangrene of these may be the cause and not the effect of the successive changes in the coagulated cruor. 11. By the employment of complete and intermitting compression, the practitioner may cure his distant patient in two visits. At the first he teaches him how to make compression himself; and at the second visit, made two or three weeks afterwards, accompanied by an assistant, he makes complete and continuous compression during a few hours, which will suffice to complete the commenced solidification, if it be not already completed. 12. No application should be made to the limb, which must be laid in a comfortable position. Compression in certain cases sufficing alone, may in others be aided by Valsalva's preparatory treatment, which, if superfluous in many cases, may be highly necessary in others. In the *Annales d'Oculistique*, November, 1858, is a translation of an interesting paper by Professor Gioppi, of Padua, in which, after taking a survey of the present state of our knowledge concerning aneurism of the ophthalmic artery, he relates a case in which digital compression proved completely successful.—(*London Medical Times and Gazette*, March 19, 1859.)

3. A Contribution to the Statistics of Cancer; Collected from the Records of the Middlesex Hospital. By *Septimus W. Sibley, Esq.*, Lecturer on Pathological Anatomy at the Hospital.—This communication is the result of an examination of 519 cases of cancer, together with the records of 172 post-mortem examinations. The more recent cases had been reported with uniformity and with some degree of fullness; some of the older cases were less perfect. In the first place the diseases embraced within the limits of the paper are defined, and what had been excluded from consideration. A table is then given, in which the seat of the primary cancer in each of the 519 cases is exhibited. There were 103 instances in the male, and 416 in the female; among the latter there were 191 of the breast, and 156 of uterine cancer.

The ages of the patients are next stated. There were three examples under the age of ten, (all males,) and one between the age of ten and twenty. Tables are given, in which the ages are arranged in decennial periods, the cases of breast and of uterine cancer being placed in separate groups. The average age of those attacked with uterine cancer was 43·28 years; with breast cancer, 48·6.

Effect of marriage, pregnancy, etc.: Of the female cancer patients, 83 per cent. either were or had been married, and among the single women the disease occurred oftener in the breast than in the uterus. Of the married women, 86 per cent. of the uterine, and 74 per cent. of the patients with breast cancer, had borne children. The average number of the births was 5·2 among the former, and 3·89 among the latter. The interval between the last pregnancy, and the proportion attacked before and after the cessation of the catamenia, are also given.

The duration of life (from the first discovery of the disease) in patients who had not been operated on, varies greatly in the different classes of cases. In the breast it is 32¼ months; in the uterus, 14; in the stomach, 8½; in the rectum, 34; in the lip, face, etc., 53; in the penis, 34½; in the bones, 10; in the labium, 29. These figures are not perfectly comparable, as in some cases, especially the external cancers, the period given is the entire duration of the disease, while in others (as in the stomach) the period is only that during which the symptoms were present.

An account is then given of the operations (by the knife) in cases of cancer of the breast. Three patients out of 60 died from the effects of the operation. The average duration of life of those who were operated on, was 53·2 months. In comparing this with the duration of life in cases in which the disease was allowed to run its natural course, (35·25 months,) it should be remembered that the cases submitted to operation are more or less selected cases.

As to the hereditary nature of the affection, the difficulties in obtaining accurate information upon this point are first alluded to. The chief of these is the very imperfect knowledge which most people, but more especially hospital patients, possess of the diseases to which their relatives have been subject. Out of 305 cases, in which the point had been particularly inquired into, 34 patients remembered to have had a relation affected with cancer. A table is given of the seat of the disease in each of the 34 cases, in 17 of which the breast was the part affected. Tables are also given, in which the degree of relationship of the cancerous relative is shown, and also the proportion affected on the father's and on the mother's side. Out of the 34 cases, in six, more than one relative was cancerous; and in one instance (the chief features of which are mentioned) no less than five relations suffered from cancer.

The existence of phthisis in different members of a cancerous family is also adverted to. This disease existed in 50 families out of 130. Similar tables to those before mentioned are given, in which the degree of kinship is exhibited; it being also noted whether the disease was on the father's or the mother's side.

The notes of the 172 post-mortem examinations are next analyzed. In the first place, a table is given, in which the seat of the primary cancer in each instance is exhibited. The cases are then arranged in the following groups: 1. Cancer of the breast. 2. Cancer of the uterus. 3. True cancer of other organs. 4. Epithelial cancer. A series of tables follows, in which the secondary cancers are enumerated, and the cases arranged as follows: *a.* The disease strictly local. *b.* Involving also the lymphatics of the part. *c.* Involving the lungs and other parts, the liver being unaffected. *d.* The liver cancerous, the lungs being free from this disease. *e.* Those cases in which there were tumors in distant parts of the body, but both the lungs and liver were free from the disease. Moreover, in each form of the affection, a list of the non-cancerous diseases found in the bodies of the cancer patients is appended.

The bearing of the foregoing facts on the mode in which cancer is disseminated throughout the body is next alluded to, three distinct modes of multiplication being recognized: 1st, the growth of tumors in the immediate neighborhood of the cancer; 2d, the development of cancer in the lymphatics of the part; 3d, the formation of cancerous tumors in distant parts of the body.

In regard to the cachexia, it was noticed that this condition only became developed as the ulceration and sloughing extended, and could not be attributed to pre-existing changes in the condition of the blood of the patient. In nearly all instances the patient died from the ordinary effects of ulceration, or from the interference with vital functions.

Dr. Webster believed the disease was much more frequent among females than males. It was a very fatal disease in this country, the average number of deaths yearly in England and Wales being about 6000. The general proportion was two females to one male, but the proportion of females in the metropolis was somewhat greater. He inquired if chimney-sweeper's cancer was less frequent in the present day than in former years, in consequence of recent legislation prohibiting the ascent of chimneys by young boys?

Mr. Hale Thomson inquired whether the author had the slightest knowledge of, or belief in, any cure for cancer? as he (Mr. Thomson) had not. The cancer might be temporarily removed by the knife; but in his experience the disease had always returned.

Mr. Arnott observed that the author had collected his facts with the greatest care, and the results he had arrived at were of the greatest importance to the profession. It had been a common idea that cancer occurred much more frequently in barren than in unmarried women. Mr. Sibley's investigations had set the matter at rest, by showing that a very small proportion of the patients in whom the disease occurred, were barren. The author had also mentioned another important fact, namely—that in cases of cancer of the mamma, the patients in whom the operation of extirpation had been performed, lived two years longer than those in whom the disease had been allowed to run its course. The general opinion, however, had been that the operation accelerated the disease, and favored its rapid progress. Another point of much importance was the number of cases in which the disease was found to be purely local, one-half of the whole number having been shown to be of that character.

Mr. Birkett had been struck with the small number of cases collected by the author in which an operation had been performed, compared with the large number of patients not operated upon. He had had it in contemplation to bring before the Society the results of an examination of a very large number of cases; and rather than make a selection in the ordinary way, which might lead to error, he intended to take the first hundred cases that died under his own observation, upon whom no operation had been performed, and then the next hundred that died upon whom an operation had been performed. The latter were, of course, to a certain extent, selected cases, because no surgeon would at once operate in a case which manifested appearances unfavorable to success; yet in many of the cases the operation could hardly be expected to prove of great ultimate benefit, and was only intended to remove a great source of local trouble and misery from the suffering patient. The average length of life in the cases in which an operation had not been performed, was three years and a half, and in the cases where an operation had been performed, it was four years and a half; thus giving the patient operated upon the benefit of one year's duration of life. He had also found that those upon whom the operation had been performed, were benefited in another respect, inasmuch as although the disease returned, it did not return with the same virulence in the part primarily affected. In some few cases the patients were relieved from any local disease at all. In the cases quoted by Mr. Sibley, the duration of life was much shorter than in those that had come under his own observation. One of the patients who died had been the subject of cancerous development, primarily in the breast, and extending over the body, upwards of thirty-two years. She complained that her first surgeon did not remove the disease, but she lived for twenty years without any trouble or annoyance. Another case lasted thirty-two years from the development of the primary growth. The subject was operated upon by Mr. Key, and was free from disease for twenty-five years. She then went to the hospital with disease on the opposite side, and died in about three years. In another case the patient lived about twenty-eight years after the primary growth was removed. Out of the three hundred patients whom he had known to die of cancers, he did not believe there were more than four or five who had not had a secondary development. The mortality arising from operations, he believed, was very much over-estimated: it was supposed to be above 10 per cent.; but in the hospital with which he was connected, it certainly did not reach 5 per cent.

The president, in answer to one of Dr. Webster's questions, stated that the Act respecting chimney-sweeps was limited to children from ten to thirteen years of age, whereas epithelial cancer did not present itself till a later period; so that the disease was not affected by the Act in question. So far as his own observation went, there had been no diminution of epithelial cancer within the last fifteen years.

Mr. Sibley regretted the smallness of the number of his cases in which operations had been performed. The records of such cases were often incomplete, as patients were lost sight of after leaving the hospital. The number of cases in which the records were complete, was sixty. There could be no error, however, in comparing a large number of cases of patients who died without an operation, with a smaller number in which the operation was performed, the result only being that in a larger number the average would be more correctly ascertained.

Mr. Moore observed, on referring to a portion of Mr. Sibley's paper, he found it stated that of the author's 519 cases, 103 occurred in males, and 416 in females.—(*London Lancet*, March 19, 1859.)

4. RECENT STRABISMUS CURED BY REMOVAL OF NECROSED BONE FROM A WHITLOW. (Under the care of *Mr. Hulke.*) The influence of peripheral sources of irritation upon the nervous system is well known. Teething and convulsions, lumbrici and epilepsy, are familiar examples as regards the great centres themselves. Instances of the sympathetic affection of single nerves, or of isolated groups of muscles, although less frequent, are by no means uncommon. We mentioned, two months ago, a case confirmatory that tænia in the intestine sometimes cause a circumscribed patch of alopecia on the scalp, and a severe but localized headache. Strabismus in young children sometimes supervenes, apparently in connection with worms. The subjects of the curious manifestations of nervous sympathy are, however, almost always either children or delicate women, in whom the cerebro-spinal system is peculiarly susceptible. The following case exemplifies the occurrence of a squint, in an adult man, seemingly in consequence of the irritation caused by a whitlow. At any rate, the squint came on coincidently with the necrosis of the terminal phalanx of one finger, and was wholly cured by the removal of the dead bone.

Owen Jones, aged thirty-seven, applied at the Royal London Ophthalmic Hospital, September 24, 1858, for advice about his left eye. He gave the following account of his case:—

Seven weeks previously, a whitlow came on the last phalanx of the thumb of his left hand; this was followed by inflammation of the lymphatics, and the gland above the elbow and those in the armpit became enlarged and very tender. The whole arm was very painful. While the limb was in this condition, one morning, on getting up, he found that he saw double, and had a squint in his left eye: this induced him to come to the Ophthalmic Hospital. It was found that he had complete paralysis of the ext. rectus muscle, and could not turn the eye out beyond the middle line. He also complained of severe circumorbital pain. He was directed to poultice the whitlow, which had burst; to foment his arm, and keep it up in a sling, on the supposition that the pain about the orbit and the paralysis of the outer rectus might depend on periosteal inflammation. Iodide of potassium was prescribed, which he took till November 23d, without any improvement of the eye. The enlargement of the glands in the armpits and above the elbow, and the swelling of the arm, had quite subsided. A loose bit of dead bone could be felt through a sinus on the under surface at the end of the thumb. It was extracted with a pair of dressing forceps.

30th.—He states that all pain in the arm, and about the orbit, left him soon after the removal of the dead bone. The squint also disappeared. He can turn the eye out till the margin of the cornea nearly touches the canthus. It is only when he looks to his extreme left that he sees double.—(*Medical Times and Gazette*, January 29, 1859.)

